# Editorial: Impact of Early Life Nutrition on Immune System Development and Related Health Outcomes in Later Life

**DOI:** 10.3389/fimmu.2021.668569

**Published:** 2021-03-25

**Authors:** Laxmi Yeruva, Daniel Munblit, Maria Carmen Collado

**Affiliations:** ^1^ Arkansas Children’s Nutrition Center, Little Rock, AR, United States; ^2^ Department of Pediatrics, University of Arkansas for Medical Sciences, Little Rock, AR, United States; ^3^ Arkansas Children’s Research Institute, Little Rock, AR, United States; ^4^ Department of Pediatrics and Pediatric Infectious Diseases, Institute of Child’s Health, Sechenov First Moscow State Medical University (Sechenov University), Moscow, Russia; ^5^ Inflammation, Repair and Development Section, National Heart and Lung Institute, Faculty of Medicine, Imperial College London, London, United Kingdom; ^6^ Solov’ev Research and Clinical Center for Neuropsychiatry, Moscow, Russia; ^7^ Department of Biotechnology, Unit of Lactic Acid Bacteria and Probiotics, Institute of Agrochemistry and Food Technology, National Research Council (IATA-CSIC), Valencia, Spain

**Keywords:** human milk, immunity, infants, metabolites, human milk oligosaccharides, microRNAs, microbiota, allergy

## Early Life Nutrition and Health Outcomes in Later Life

Human milk (HM) is a complex mixture of macronutrients and bioactive compounds that provide optimal nutrition to infants (1–5). HM has been shown to impact infant’s gastro-intestinal tract, immune system, microbiota composition, metabolism and also may have long-term effects on the development of infectious and non-communicable diseases (3, 6–8). The aim of this editorial is to provide a summary of the original research, reviews and opinions regarding key factors affecting human milk composition, and the role of bioactive components of human milk on infants’ health.

Maternal obesity and maternal atopy are highly prevalent states that may have an effect on HM composition and infants’ health outcomes (9–14). Few studies, however, have attempted to evaluate associations between HM metabolome composition and measures of infants’ health and development. For instance, Bardanzellu et al. reviewed different studies for HM metabolite profile from mothers with overweight and obesity in an attempt to determine the milk metabolome composition with respect to obesity. However, the small sample size and large variability of the measures precluded the investigators from drawing conclusions which underscores the necessity of large sample size studies in this area of research. The authors, however, found that the fatty acid profile of human milk was associated with the maternal obesity status. Specifically, higher levels of saturated fatty acids and lower levels of monounsaturated and n-3 long-chain polyunsaturated fatty acids were found in milk of women with obesity compared to milk of women with normal weight. These changes in milk composition may influence long-term weight gain and glucose tolerance, in infants.

Allergic diseases are of a major concern and a significant burden to healthcare. It has been previously shown that HM composition may differ in allergic and non-allergic mothers (15). Recent research from Stinson et al. demonstrated that human milk from atopic mothers had lower levels of short-chain fatty acids (SCFA). Importantly, reduced levels of SCFA during early life may program the gut, microbiota, and obesity in infants. Nutritional interventions during pregnancy and lactation could serve as strategies to mitigate maternal atopy and potentially improve HM composition. For instance, Kao et al. showed that maternal consumption of goat milk during pregnancy and lactation associated with reduced airway inflammation and allergy outcomes in the offspring compared to cow’s milk consumption. The goat milk feeding had increased immunoglobulin levels, Th1 cytokine production, and improved NK cell activity in comparison to cow’ milk feeding in the offspring. In addition, in an animal study by Adel-Patient et al. showed that altering maternal immune status by sensitizing to different antigens protects offspring by modulating the antibody composition of human milk to specific antigens. In summary, obesity and prenatal antigen exposure of mothers were associated with HM composition and may affect infant health and development, but relationships should be confirmed in methodologically rigorous studies with a large sample size.

Human milk feeding likely protects from pathogens, thereby reducing/preventing negative outcomes associated with infection *via* different bioactives of milk such as human milk oligosaccharides (HMOs) and free amino acids (FAAs) (1, 16–19). Indeed, Carr et al. review highlighted the antipathogenic and immunomodulatory properties of HMOs and Zuurveld et al. reviewed the potential role of HMOs in preventing allergic diseases. In their article Sadelhoff et al., discuss the potential role of amino acids (particularly glutamine and glutamate) in HM to protect against neonatal allergies and infection. Further, using a HM-fed piglet model, Rosa et al. demonstrated the appearance of HM metabolites’ in the gut, serum, and urine of HM-fed piglets. Importantly, glutamic acid and glutamate levels were higher in the HM-fed animals relative to the formula fed group suggesting potential benefits of HM FAAs. Also, Rosa et al., study discussed human metabolites such as polyamines and tryptophan impact on immune response.

Human milk has been shown to promote gut microbiota development and function (20–25). In reviewing the literature, Carr et al. comprehensively overviewed the role of HM microbiota on gut microbiota colonization and immune function. This article also discussed the role of human milk components such as HMOs, and IgA impact on gut microbiota. Peroni et al. reviewed the literature regarding microbiome composition and its impact on the development of allergic diseases. Drall et al. demonstrated an association of microbiota composition in exclusively breastfed infants to *C. difficile* colonization. In summary, dietary intake and both pre- and post-natal factors appear to be associated with the gut microbiota composition and its association to pathogens colonization. This may be a focus for the future intervention strategies aiming at improving infants health.

Previous studies suggest antipathogenic effects of HM components and that the addition of these bioactive molecules (i.e., HMOs, lactoferrin, immunoglobulins, and milk fat globule membrane FGM, extracellular vesicles) to infant formulas may benefit child health (20, 26–36), although the studies usually lack methodological rigor and outcomes were based on a small sample size. The studies on recombinant immunoglobulins and bioactivity in the digestive tract are limited. Research from Sah et al. provided some evidence that recombinant antibody towards respiratory syncytial virus (RSV) may impact growth and have neutralization activity against the virus across the GI tract. In another study, Nederend et al. demonstrated that bovine immunoglobulin antiviral activity and T cell response may prevent RSV infection. Interestingly, Adel-Patient et al. found no protection to protein present in cow’s milk by feeding the hydrolysates of caseins and *Lactobacillus rhamnosus* GG protobiotic. Thus, future studies are needed to fully understand the protective effects of immunoglobulins, as well as pre and probiotics, before adding these components to infant formula. The combined effect of different bioactive molecules within the formula on infant health and development also requires further investigation.

Human milk may impart benefits through epigenetic programming influencing long-term health by various mechanisms. van Esch et al. provided an overview on the evidence of maternal nutrition, environmental factors impact on milk composition, and how the different components of milk epigenetically program infants’ health and dictate allergy and asthma outcomes in later life. Human milk contains extracellular vesicles with microRNAs (miRNAs) as one of the epigenetic molecules (35). Furthermore, Carr et al. provided evidence that miRNAs known to modulate gene expression were associated with immune function in the human milk-fed group compared to formula diet-fed group in the piglet model. Also, the review by Carr and associates highlighted that miRNAs present in human milk may be associated with a beneficial effect for infants’ health and immune system.

Finally, Bilsen and colleagues elegantly show how a network-based approach that includes evidence from studies to determine the windows of opportunity to shape lifelong health of infants. This can be used to predict the key candidate markers of early life immune development. Human milk is a complex mixture with several bioactive components providing short and long-term health benefits to infants. We sincerely hope that the article’s compilation of the Research Topic on human milk will be useful and interesting to the readers and hope that the knowledge gaps highlighted will be considered for future state-of the art research findings.

## Author Contributions

All authors listed have made a substantial, direct, and intellectual contribution to the work and approved it for publication.

## Funding

LY is supported by USDA-ARS Project 6026-51000-012-06S, and by NIH 1R21AI146521.

## Conflict of Interest

The authors declare that the research was conducted in the absence of any commercial or financial relationships that could be construed as a potential conflict of interest.
